# Rapid Detection and Immune Characterization of *Mycobacterium abscessus* Infection in Cystic Fibrosis Patients

**DOI:** 10.1371/journal.pone.0119737

**Published:** 2015-03-05

**Authors:** Mathis Steindor, Vanesa Nkwouano, Ertan Mayatepek, Colin R. Mackenzie, Dirk Schramm, Marc Jacobsen

**Affiliations:** 1 Department of General Pediatrics, Neonatology, and Pediatric Cardiology, University Children’s Hospital, Heinrich Heine University, 40225 Duesseldorf, Germany; 2 Institute of Medical Microbiology and Hospital Hygiene, Heinrich Heine University, 40225 Duesseldorf, Germany; National Institute of Infectious Diseases, JAPAN

## Abstract

Cystic fibrosis patients are highly susceptible to infections with non-tuberculous mycobacteria. Especially *Mycobacterium abscessus* infections are common but reliable diagnosis is hampered by non-specific clinical symptoms and insensitive mycobacterial culture. In the present study we established novel methods for rapid detection and immune characterization of *Mycobacterium abscessus* infection in cystic fibrosis patients. We performed *Mycobacterium abscessus* specific DNA-strip- and quantitative PCR-based analyses of non-cultured sputum samples to detect and characterize *Mycobacterium abscessus* infections. Concomitantly *in vitro* T-cell reactivation with purified protein derivatives (PPDs) from different mycobacterial species was used to determine *Mycobacterium abscessus* specific T-cell cytokine expression of infected cystic fibrosis patients. Four of 35 cystic fibrosis patients (11.4%) were *Mycobacterium abscessus* culture positive and showed concordant DNA-strip-test results. Quantitative PCR revealed marked differences of mycobacterial burden between cystic fibrosis patients and during disease course. Tandem-repeat analysis classified distinct *Mycobacterium abscessus* strains of infected cystic fibrosis patients and excluded patient-to-patient transmission. *Mycobacterium abscessus* specific T-cells were detected in the blood of cystic fibrosis patients with confirmed chronic infection and a subgroup of patients without evidence of *Mycobacterium abscessus* infection. Comparison of cytokine expression and phenotypic markers revealed increased proportions of CD40L positive T-cells that lack Interleukin-2 expression as a marker for chronic *Mycobacterium abscessus* infections in cystic fibrosis patients. Direct sputum examination enabled rapid diagnosis and quantification of *Mycobacterium abscessus* in cystic fibrosis patients. T-cell *in vitro* reactivation and cytokine expression analyses may contribute to diagnosis of chronic *Mycobacterium abscessus* infection.

## Introduction

Mutations on both alleles of the Cystic Fibrosis Transmembrane Conductance Regulator (CFTR) are the genetic cause of Cystic Fibrosis (CF), the most common single-gene caused disease in Caucasians [1]. CF pathology affects multiple organs, but pulmonary disease predominantly influences morbidity and mortality of CF patients. Chronic pulmonary infections are a typical feature of CF [2]. Non-tuberculous mycobacteria are rarely pathogenic for immunocompetent individuals but frequently colonize vulnerable pulmonary epithelia of CF patients [3]. Closely coherent extrinsic factors (e.g. frequent infection with other opportunistic bacteria, viruses, and fungi) and intrinsic factors (e.g. continuous inflammation, dysregulation of innate and adaptive immune response) contribute to increased susceptibility against non-tuberculous mycobacteria but the exact mechanism remains elusive [4, 5].

*Mycobacterium (M.) avium complex* (MAC) and *M. abscessus complex* (MABSC) are the non-tuberculous mycobacterial species most commonly detected in the sputum of CF patients [6]. MABSC has recently been classified as an independent species [7]. Since then, MABSC has been found to be the most frequent ‘rapid growing’ human pathogenic mycobacterial species [8]. MABSC shares some similarities with the highly pathogenic species of the *M. tuberculosis complex* (MTC). Most intriguingly MABSC is able to cause persistent lung disease characterized by development of caseous lesions, a hallmark of human tuberculosis [9]. Recently evidence for direct transmission of MABSC between CF patients has been found [10]. Although the mode and likelihood of patient-to-patient transmission of MABSC is unclear, this finding will have major implications for clinical routine. In addition, the possibility of direct transmission renders early detection and characterization of MABSC in CF patients crucial.

The reported prevalence of MABSC infections in CF patients differs markedly between studies of different regions ranging from 3.4 to 24% [3]. These differences are at least in part due to difficulties in the diagnosis and detection [11]. A few major factors contribute to this divergence. First, clinical symptoms of MABSC infections are shared with several other infections and imaging methods are often inconclusive [11]. Second, mycobacterial culture from pulmonary samples (predominantly sputum) is time consuming and fails in many cases primarily due to fast growing colonizing bacteria such as pseudomonas and staphylococci [12]. Third, decontamination of sputum samples, a prerequisite for detection of mycobacteria, reduces the sensitivity for detection of mycobacteria [13]. Immunological tests for MABSC infections are not available and cross-reactivity of immune responses against different mycobacteria hampers the development of specific assays [14]. Previous studies have used purified protein derivatives of MTC or MAC for skin tests or *in vitro* assays to discriminate between different mycobacterial infections with encouraging results [15].

In the present study we established PCR-based methods (i.e. DNA-strip test, quantitative PCR) for rapid *ex vivo* detection and quantification of MABSC from sputum of CF patients. A T-cell assay based on *in vitro* reactivation with different mycobacterial antigens was performed to distinguish and characterize immunity to mycobacterial infections. This approach may help to define immune characteristics that lead to increased susceptibility of CF patients against non-tuberculous mycobacteria and may improve early diagnosis of infection.

## Methods

### Study subjects and design

35 patients diagnosed with CF were recruited in the Department for General Pediatrics, University Children´s Clinic, Duesseldorf and enrolled in the study in 2013 (starting in March). Clinical characteristics of the CF patients are reported in Table 1. Heparinized blood (3 ml) and sputum samples were collected as part of routine evaluation. Routine culture for detection of non-tuberculous mycobacteria has been performed for all CF patients able to expectorate sputum samples. From one CF patient, sputum and blood samples were taken consecutively over a period of 200 days. Children unable to expectorate sputum donated peripheral blood samples. Written informed consent was obtained from all subjects or their guardians.

**Table 1:** Clinical characteristics of CF patients

	CF patients
number	35
Gender distribution (f/m)	18/17
Age mean (range)	13,1 (3–25)
FEV1% mean (range)	78,2 (37,2–124,1)
BMI mean (range)	17,9 (12,3–27)

n: number of patients; f/m: number of females/males; FEV: forced expiratory volume; BMI: body mass index

### Ethics Statement

This study was approved by the ethics committee of the University Hospital Duesseldorf (Internal Study No. 4505).

### MABSC-DNA-strip test

Sputum samples were immediately processed after expectoration or stored at -20°C for up to 60 days. A minimal volume of 50 μl was used for DNA isolation. DNA was extracted using the enzymatic lysis-based InnuPrep Mycobacteria DNA Kit (Analytik Jena). The DNA-strip test was performed using GenoType Mycobacterium CM kit (Hain Lifescience) following the manufacturer’s protocol. To adjust for estimated lower mycobacterial numbers (as compared to culture enriched mycobacteria), we modified PCR-settings by adding ten cycles.

### Quantitative PCR (qPCR) for MABSC *rpoB*

TaqMan (Life Technologies) real-time PCR for the MABSC *rpoB* gene was performed. A region in the *rpoB* gene specific for MABSC (not present in *M. chelonae*) was targeted [16]. Primers and probe (Table 2) were designed using Primer Express (Applied Biosystems). To evaluate assay specificity we tested following mycobacteria strains (i.e. *M. chelonae*, *M. kansasii*, *M. gordonii*, *M. fortuitum*, *M. szulgai*, *M. marinum*, *M. avium*, *M. intracellulare*, *M. celatum*, *M. simiae*, *M. malmoense*) from patient isolates. None of these related mycobacteria strains were detected. As internal standards, plasmids containing the *rpoB*-PCR target sequence were generated and added to each experiment. In brief, *rpoB* amplicons were ligated in plasmids and transformed to DH5α-*E.coli* using pGEM-T-Vector Kit (Promega) and isolated using the High Pure Plasmid Isolation Kit (Roche). Plasmid concentration was measured in a Nanodrop 1000 spectrophotometer (Thermo Scientific) and calculated plasmid concentrations of 10^5^ and 10^2^ copies were used as standards. Sputum DNA samples (5 μl) were used for each qPCR performed according to manufacturer’s instructions (QuaniTect Multiplex PCR NoROX, Qiagen) on a C1000 Thermocycler (Biorad). For MABSC spike-in experiments, sputum of a MABSC-negative CF patient was spiked with serial dilutions (10-fold) of a MABSC patient isolate. CFU were then determined in triplicates after 72h incubation on sheep-blood-agar. Concomitantly DNA-strip tests and qPCR were performed for spiked sputum samples (as described above).

**Table 2:** Primers and probe used for qPCR

Target	Accession No.	Primers and probe
*rpoB*	AY147164.1	3635(+)CGATAGAGGACTTCGCCTAACC
		3711(-)TCGAGCACGTAAACTCCCTTTC
		3660(+)HEX-CCACTGACCGAACATCTATCCCGC

### Variable number tandem repeat (VNTR) analysis

Classification of culture-enriched MABSC from sputum was performed by variable number tandem repeat (VNTR) analysis according to Wong *et al*. [17] with minor modifications. In brief, six of the described tandem repeats (TR45, TR109, TR116, TR150, TR155, TR172) were used for PCR analysis and tandem-repeat lengths were analyzed using agarose gel (1%) electrophoresis.

### Flow cytometry-based detection of MABSC-specific T-cells

Heparinized blood was processed freshly (within five hours of blood collection) to assure maximal sensitivity of this assay (own unpublished data). In brief, blood (100 μl) was diluted (1:1) in RPMI containing 1% L-Glutamine (Sigma Aldrich), 1% Penicillin/Streptomycin (Life Technologies) and supplemented with recombinant human (rh) IL-7 (20 ng/ml; Biolegend) for overnight culture in 96-well round-bottomed microtiter plates as described [15]. IL-7 has been shown previously to increase the sensitivity of IFNγ release assays (IGRAs) for detection of mycobacteria specific T cells [18]. IL-7 has a minimal cytokine-inducing effect in the absence of antigens. The following antigens were used for stimulation: purified protein derivative (PPD) of MABSC (termed abscessin), PPD of *M. avium/intracellulare* (sensitin), and PPD of *M. tuberculosis* (tuberculin). All PPDs were purchased from Statens Serum Institute. *Staphylococcus* enterotoxin b (SEB) (15 μg/ml, Sigma Aldrich) was used as a positive control. The following monoclonal fluorescence-labeled antibodies were used: CD4 (clone Okt4, BV510), CD45RA (clone HI100, FITC), CCR7 (clone 3D12, PE-Cy7), IFNɣ (clone B27, BV450), TNFα (clone Mab11, APC), IL-2 (clone MQ1–17H12, PerCP-Cy5.5), and CD40L (clone 24–31, PE). All antibodies (except CCR7: BD Biosciences) were purchased from BioLegend. A FACS Canto-II flow cytometer (BD Biosciences) was used for the measurements and FCS Express 4 Software (De Novo) was used for analyses. The background values [cytokine positive T-cell proportions of non-stimulated (rhIL-7 only) samples] were subtracted from each stimulus. Ratios of PPD specific T-cells were calculated by dividing the proportions of CD40L/IL-2 double positive T-cell proportions for abscessin/tuberculin and abscessin/sensitin. For calculation of normalized values, we set the sum of abscessin-, tuberculin-, and sensitin-specific CD40L/IL-2 double positive T-cells proportions to 1. For presentation of cytokine expression pattern (i.e. three cytokines and CD40L) graphical depiction is not possible because 4-dimensional presentation (followed by selection of subsets) is technically not feasible. As a consequence the events for each cytokine pattern expressing T-cell subpopulation (numbers of non-stimulated numbers were subtracted) were counted and the number of T cells expressing at least one cytokine per activation marker in response to *M. abscessus* was calculated. Calculated proportions of each subpopulation of all activated T cells are indicated for each individual donor and compared between study groups.

### Statistical analyses

Statistical calculations were performed using SigmaStat (Systat Software). Parametric or non-parametric tests were chosen according to Kolmogorov-Smirnow normality test. Accordingly, the student t-test or the Mann-Whitney Rank Sum Test was used and indicated in the text and figure legend. *P*-values < 0.05 were considered to be significantly different.

## Results

### Direct sputum DNA-strip/*rpoB*-qPCR MABSC tests

**Fig 1:**
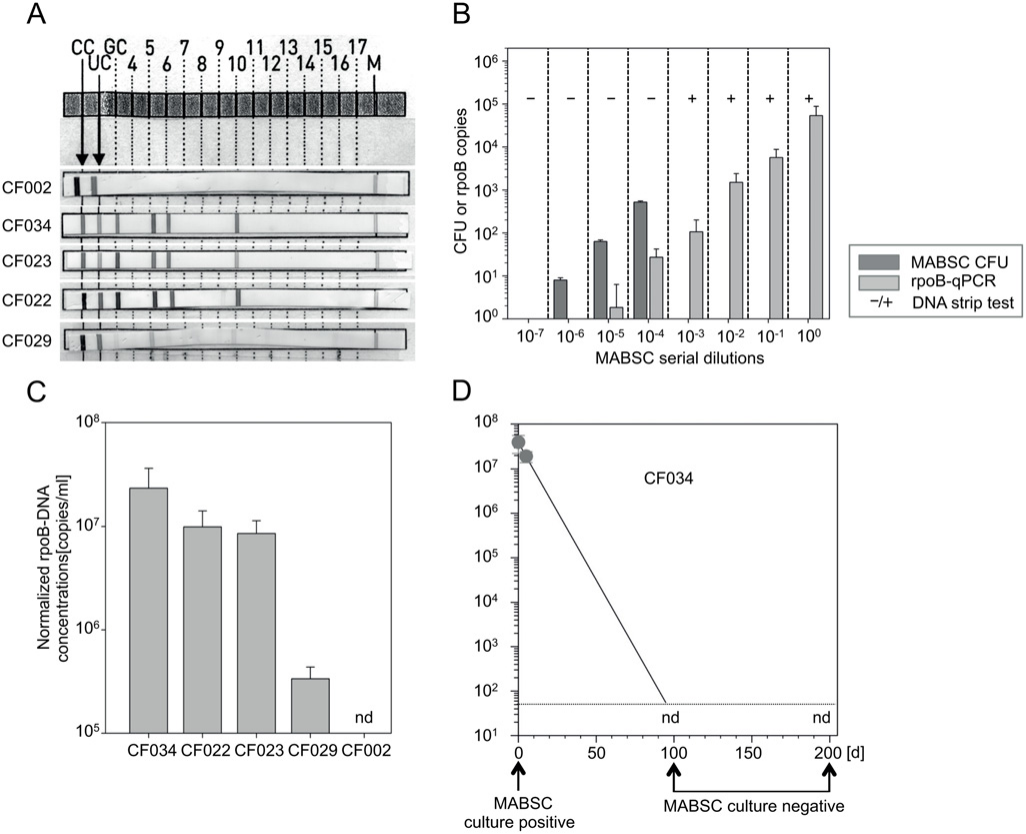
Direct detection and quantification of MABSC by CFU, DNA-strip, and rpoB-qPCR of non-cultured CF sputum samples. (A) Direct *ex vivo* PCR strip results of sputum from MABSC-positive CF patients (i.e. 034, 023, 022, 029) and CF patient 002 (previous MABSC infection but unclear infection status). A band pattern at positions 3, 5, 6, and 10 indicates MABSC (the same pattern but absence of a band at position 3 and 6 indicates either *M. abscessus* or *M. chelonae*). The CC band is a conjugate control, the UC band indicates bacterial DNA with a high GC-content, the GC band indicates DNA of the genus *mycobacterium*. (B) CFU (colony forming units), *rpoB*-qPCR (both at the y-axis), and PCR strip test results (positive tests indicated as +, negative as-) for serial dilution (10-fold) of MABSC in sputum. Dark grey bars indicate CFU numbers. Bright grey bars indicate normalized *rpoB* copies. Mean values of triplicate samples and standard deviations are given. One representative experiment of two is shown. (C) *RpoB*-qPCR results of sputum from MABSC-positive CF patients (i.e. 034, 023, 022, 029) and CF patient 002 are depicted. Calculated MABSC sputum concentrations (adjusted for 50-fold lower sensitivity of rpo-qPCR as compared to MABSC culture) are given. (D) Time course analyses of *rpoB*-qPCR and mycobacterial sputum culture from CF patient CF034. Circles depict *rpoB*-qPCR values and arrows indicate time points of MABSC sputum culture. nd (not detectable) indicates *rpoB*-qPCR runs where MABSC *rpoB* was not detected. The dotted line indicates the detection limit of qPCR.

Mycobacterial sputum culture detected MABSC infection in four of 35 enrolled CF patients (11.4%) at study onset. We performed a DNA-strip test on sputum without prior *in vitro* culture from all sputum expectorating CF patients (n = 23). DNA-strip test detected MABSC in the sputum of all culture positive CF patients (CF034, CF023, CF029, CF022) (Fig. 1A, Table 3). None of the culture-negative CF patients had a positive PCR and CF002, a patient with a history of MABSC infection, was also DNA-strip test negative (Fig. 1A and Table 3). Next we analyzed the sensitivity of DNA-strip test in sputum samples by establishing a MABSC-specific *rpoB*-qPCR. Serial dilutions of MABSC in sputum and concomitant analyses of CFU, DNA-strip test, and *rpoB*-qPCR revealed ten CFU at the 10^–6^ dilution step (Fig. 1B). The *rpoB*-qPCR detected about two DNA copies at the 10^–5^ dilution step (about 100 CFU) (Fig. 1B). Accordingly, a rough estimate of 50-fold lower sensitivity of *rpoB*-qPCR as compared to CFU was deduced and applied for calculation of MABSC concentration in CF sputum samples. Notably, the DNA-strip test was positive at 10^–3^ but not at lower concentration. This indicated an about 100-fold lower sensitivity of the DNA-strip test as compared to *rpoB*-qPCR and a detection limit of estimated 5x10^3^ CFU/ml sputum.

**Table 3:** Study results.

	CF patients (n = 35)		
sputum samples	available (n = 23)		not available (n = 12)
MABSC sputum culture	positive (n = 4(5*))	negative (n = 18)	na
DNA-strip test (pos/neg)	4/1	0/18	na
MABSC rpo-qPCR (pos/neg)	4/1	0/18	na
MABSC immune test (pos/neg)	5/0	2/16	3/9

* One CF patient (CF002) had a history of previous MABSC infection but an unclear infection status at study onset due to persistent culture contamination. For sputum PCR analyses this patient was classified as MABSC negative (confirmed by our assays). n: number of patients; na: not applicable; pos: positive; neg: negative.

### Sputum MABSC characterization of CF patients

Next, *rpoB*-qPCR was applied to determine MABSC concentrations of CF patient´s sputum samples. MABSC-confirmed CF patients had marked differences of MABSC sputum burden with concentrations ranging from 5.6x10^5^ to 3.9x10^7^ bacteria per ml sputum (Fig. 1C). All MABSC-negative CF patients (including CF002; Fig. 1C) were *rpoB*-qPCR negative (Table 3). For one CF patient (CF034), *rpoB*-qPCR and MABSC sputum culture were performed repeatedly during the study period. Initially CF034 had the highest concentrations of MABSC in sputum (Fig. 1C, D) and was culture positive. At the next routine visits (97 and 200 days after study onset), CF034 had MABSC-negative in sputum cultures (Fig. 1D) and was also *rpoB*-qPCR negative (Fig. 1D).

**Fig 2:**
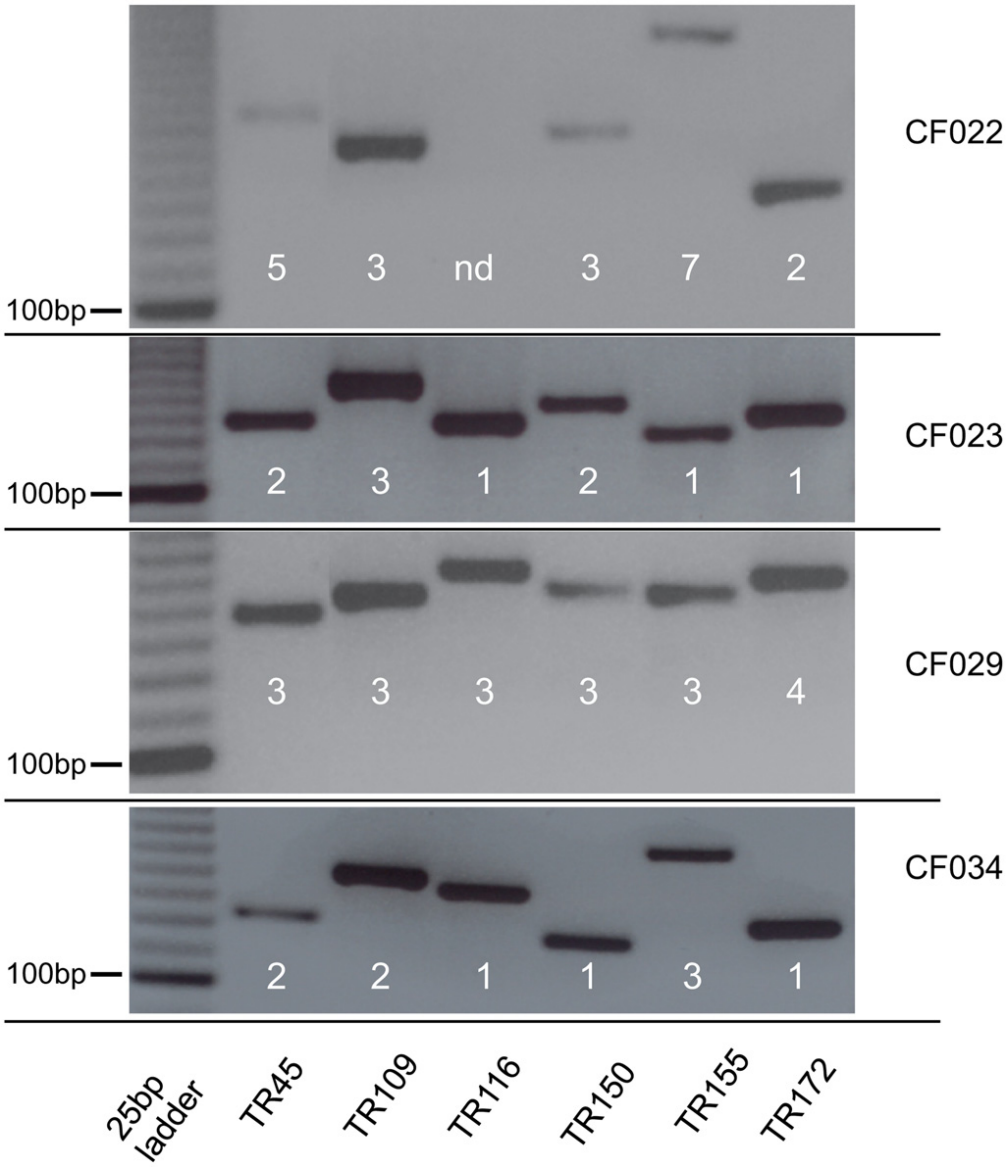
Characterization of MABSC strains from CF patients by VNTR (Variable Number Tandem Repeat) analysis. VNTR-PCR results of MABSC-confirmed CF patients are depicted. Agarose gel electrophoresis for six tandem repeat regions are shown. Differences in the band pattern indicate MABSC strain specific differences.

The high prevalence of MABSC-positive sputum samples from CF patients in the present study suggested a common source of infection or direct transmission between patients [19]. Consequently, we characterized MABSC strains from CF patients by VNTR-analysis [17]. Altogether six regions were analyzed but none of the MABSC isolates from CF patients had identical patterns (Fig. 2). These results rendered transmission of MABSC strains between CF patients unlikely. Therefore our novel approaches enabled us to detect marked inter-individual differences and changes of MABSC sputum concentrations during disease course caused by different MABSC strains.

### MABSC-specific T-cells in CF patients

**Fig 3:**
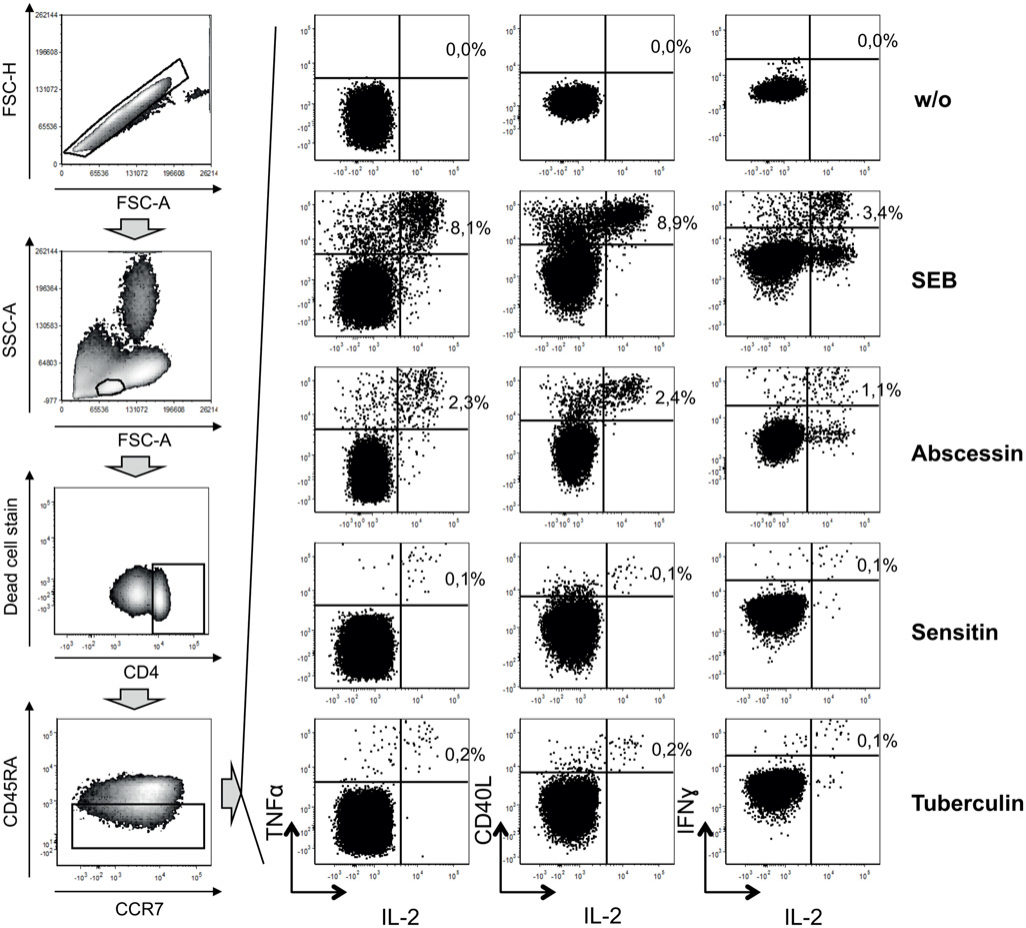
Gating strategy of cytokine-expressing T-cell proportions. Density plots (left graphs) and dot plots (right graphs) depict flow cytometry analyses of data of whole blood *in vitro* restimulation. Results of stimulation with different mycobacterial antigens, i.e. abscessin of *M. abscessus* (MABSC), sensitin of *M. avium* (MAC), and tuberculin of *M. tuberculosis* (MTB), the non-clonal T-cell activator SEB (Staphylococcus enterotoxin B), and without stimulation (w/o) are shown. Grey block-arrows indicate the sequence of analyses steps. After excluding cell doublets by comparing FSC-A (forward scatter area) and FSC-H (forward scatter height) parameters (upper left density plot), lymphocytes were selected on the basis of cellular size (FSC-A) and granularity (SSC-A; side scatter area). Viable CD4^+^ memory T-cells (CD45RA negative) were selected for cytokine analysis. Proportions of TNFα/IL-2, CD40L/IL-2, and IFNɣ/IL-2 double positive T-cells were determined for each stimuli (right graphs). A representative analysis of a MABSC infected CF patient is shown.

**Fig 4:**
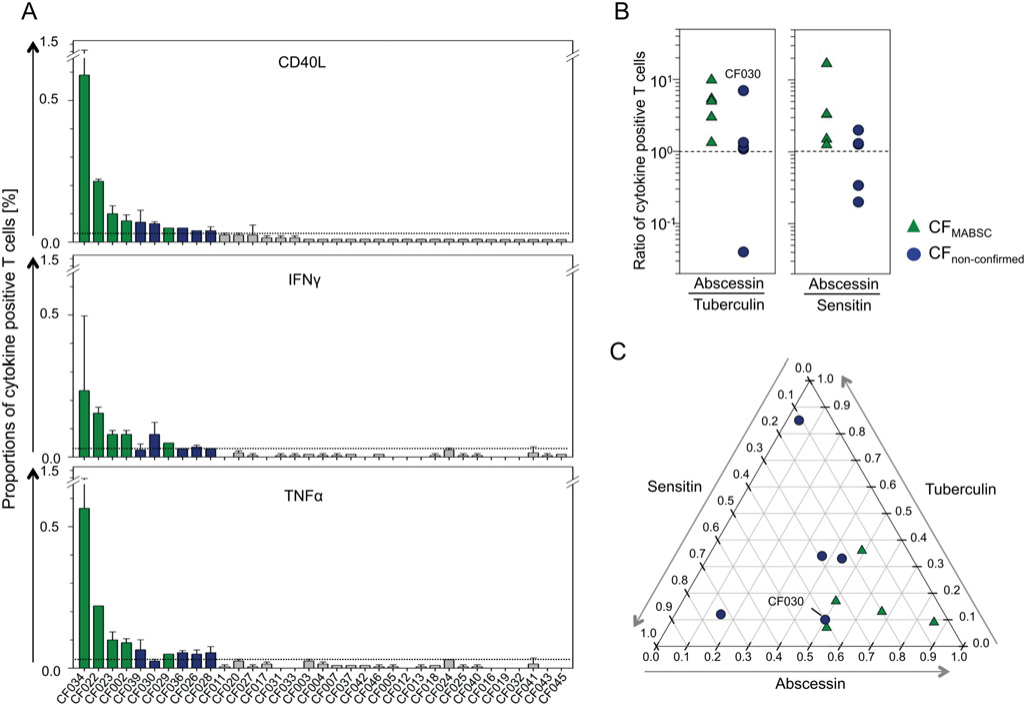
Cytokine expression of abscessin specific T-cells. MABSC induced cytokine-expressing T-cell proportions of CF patients (n = 35). (A) Abscessin specific T-cells that produce IL-2 together with either CD40L (upper graph), or IFNɣ (middle graph), or TNFα (lower graph) are shown. Bars indicate mean and standard deviation of measured duplicates for each individual patient (ID label on the x-axis). Green color indicates MABSC confirmed cases. Blue color indicates CF patients without MABSC infection (current and past) but a positive T-cell response against MABSC, defined by at least two of three cytokine combinations values [mean proportions] above 0.03% [3-times the assumed flow cytometry detection limit] (B) Comparisons of T-cell cytokine responses induced by abscessin, tuberculin, and sensitin. Ratios (abscessin / tuberculin or abscessin / sensitin) for CD40L/IL-2 positive T-cells are shown for confirmed MABSC infected CF patients (green triangles) and non-confirmed MABSC responders (blue circles). Each symbol represents ratios calculated of mean proportions for an individual donor. The dotted lines indicate equal T-cell responses (ratio = 1) (C) Relative T-cell responses against abscessin, tuberculin, and sensitin are depicted in a ternary plot. The sum of T-cell proportions for each individual donor was set to 1. Relative values of specific T-cells for each PPD are indicated in a ternary plot. MABSC infected CF patients are depicted as green triangles and non-confirmed MABSC responders as blue circles. The MABSC non-confirmed CF patient CF030 is highlighted who has 50% abscessin, 40% sensitin, and 10% tuberculin specific T-cells.

Immune-based tests for MABSC infection are not available. Hence, we established an *in vitro* whole blood assay to detect MABSC-specific T-cells using flow cytometry analysis of intracellular cytokines (for details see Methods section and Fig. 3). Ten CF patients showed positive cytokine responses to MABSC-specific PPD (abscessin) (Fig. 4A). These comprised all confirmed MABSC-infected CF patients (CF022, CF023, CF029, CF034, CF002) and five CF patients (CF026, CF028, CF030, CF036, CF039) without evidence for MABSC infection (CF_non-confirmed_) (Fig. 4A, Table 3, S2 Fig.). Three patients of the CF_non-confirmed_ patient group (CF026, CF028, CF036) were unable to expectorate sputum and therefore mycobacterial culture or PCR-based analyses were not possible. Cross-reactive mycobacterial antigens may confound these results but concomitant testing of PPDs from different mycobacteria (i.e. *M. tuberculosis*, tuberculin; *M. avium*, sensitin) was shown to reveal the causative mycobacteria [15]. Hence we compared T-cell responses between abscessin and tuberculin (Fig. 4B, left graph), as well as abscessin and sensitin (Fig. 4B; right graph), and detected higher T-cell proportions specific for abscessin (ratio > 1) for all *M. abscessus* confirmed cases (CF_MABSC_, green triangles) (Fig. 4b). In contrast, in CF patients without indication of MABSC infection (blue circles) only one patient (CF030) had a stronger abscessin specific response (Fig. 4B). In accordance, T-cell responses against all three PPDs detected more than 50% of abscessin-specific T-cells for confirmed cases (Fig. 4C). Again, only CF030 had an abscessin dominant T-cell response similar to confirmed cases. We concluded that T-cell response ratios confirmed MABSC infection and suggested non-MABSC related causes in the majority of CF_non-confirmed_ patients.

### Cytokine-expression pattern discriminate confirmed MABSC cases (CF_MABSC_)

**Fig 5:**
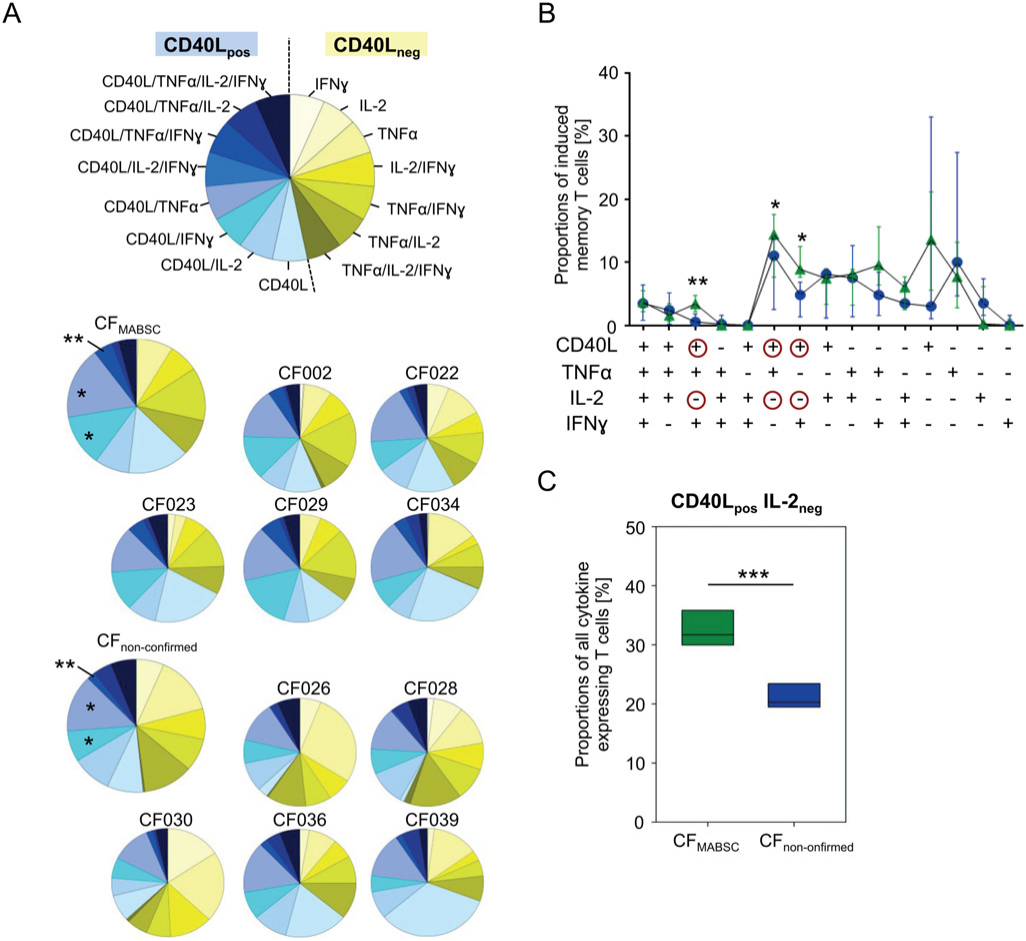
Cytokine expression patterns of MABSC-specific T-cells. Cytokine expression pattern of MABSC-specific T-cells for TNFα, IFNɣ, IL-2, and CD40L are shown. Data were normalized for each individual donor by setting the number of all cells expressing at least one cytokine to 100%. (A) CD40L positive (blue colors) and CD40L negative (yellow colors) subpopulations with different cytokine expressing subpopulations are indicated. An explanatory pie chart is depicted at the top. Pie charts represent data of individual CF patients (with the patient ID indicated above) and mean values for each study group (upper left pies). (B) CF patient groups with confirmed MABSC infection (green triangles) and non-confirmed MABSC responders (blue circles) are compared for cytokine expression pattern of MABSC-specific T-cells (i.e. quadruple, triple, double, single positive). Line and scatter plots indicate median expression levels and standard deviation (y axis) of distinct cytokine pattern expressing subsets (x-axis). Red circles highlight CD40L and IL-2 expression status for significantly different subsets. The student *t* test was used to evaluate data between study groups and significant differences are indicated as asterisks; *: *P*<0.05 **, *P*<0.01. (C) A box plot shows the sum of all CD40L_positive_ and IL-2_negative_ T-cell subsets for MABSC infected CF patients (green) and non-confirmed MABSC responders (blue). The student *t* test was used to evaluate data between study groups. Significant differences are indicated as asterisks; ***, *P*<0.001.

Cytokine-expression pattern characterize functional T-cell differences and may reflect different infection stages (e.g. acute and previous MABSC infection). Three cytokines (i.e. IFNγ, TNFα, IL-2) and the T-cell activation marker CD40L were analyzed concomitantly in abscessin-specific T-cells. Expression patterns revealed significant differences between CF_MABSC_ and CF_non-confirmed_ patients (Fig. 5A,B). CF_MABSC_ had higher levels of CD40L/IFNγ/TNFα triple-positive (p = 0.004), CD40L/IFNγ (p = 0.01), and CD40L/TNFα (p = 0.02) double-positive abscessin-specific T-cells. Notably, differences applied to T-cell populations that expressed CD40L but not IL-2 (Fig. 5B,C). Comparison of all CD40L-positive/IL-2-negative T-cell proportions revealed non-overlapping differences between CF_MABSC_ (median 32.8%, range 6%) and CF_non-confirmed_ patients (median 21.7%, range 3.3%) (p<0.001) (Fig. 5C). We concluded that increased MABSC-specific T-cell expression of CD40L in the absence of IL-2 production is an immune marker of MABSC infection in CF patients.

## Discussion

Here we characterized MABSC-specific T-cell responses in CF patients and revealed cytokine expression pattern indicative of chronic MABSC infection. In addition, novel PCR-based methods for rapid and reliable *ex vivo* diagnosis of MABSC infection in CF patients were established. DNA-strip test and *rpoB*-qPCR based detection of MABSC revealed concordant results with mycobacterial culture. This finding has potential important implications for diagnosis of MABSC infection because direct PCR-based analysis of CF sputum samples bypasses NTM culture that is time consuming and frequently fails [20]. In addition, reduced sample volumes (about 100 μl) were sufficient for DNA-strip tests whereas mycobacterial culture usually requires at least 10-fold larger volumes [21]. Thus, the DNA-strip test would be particularly important for CF patients with low sputum sample volumes (e.g. young children).

MABSC-specific *rpoB*-qPCR had a 100-fold higher sensitivity as compared to the DNA-strip test and allowed quantification of mycobacteria in sputum. MABSC sputum burden varied markedly between CF patients and initial analyses during disease course of one CF patient indicated fluctuating MABSC concentrations between 10^7^ and ‘not detectable’ low numbers (below the detection limit of estimated 50 bacteria) of mycobacteria per ml sputum. Potentially, quantification of mycobacteria in CF sputum samples may become an important diagnostic tool to monitor efficacy of challenging MABSC therapy. Since standard therapy regimes for MABSC infection are not available, such marker would be of paramount clinical relevance. Ongoing studies apply DNA-strip tests for sputum samples in enlarged CF patient cohorts to confirm reliability of the novel assays.

In our study, all patients with current or past MABSC infection had MABSC-specific and-dominant T-cell responses. This finding indicated a high sensitivity of this assay, rendering it a putative strong tool for exclusion of MABSC infection in CF patients. However, MABSC-specific T-cell responses could be observed for patients without confirmed MABSC infection as well. Indeed, immunological detection of MABSC infection may always be confounded by several environmental and population specific factors that induce cross-reactive T-cells including i) Bacille-Calmette Guérin (BCG) vaccination; ii) latent *M. tuberculosis* infection; iii) previous contact to atypical mycobacteria. Although we were able to exclude the first two factors [BCG vaccination is not performed in Germany and IGRAs were negative for CF patients with a positive T-cell response against mycobacteria (data not shown)], a previous contact with atypical mycobacteria cannot be excluded. Therefore we assume that MABSC non-confirmed CF patients underwent asymptomatic infection (e.g. colonization) with different atypical mycobacteria in the past. This assumption is supported by the heterogeneity of PPD specificity in this group as compared to MABSC confirmed CF patients that suggested different causative mycobacteria. To avoid or at least minimize mycobacterial cross-reactivity for the detection of MABSC infection, identification of MABSC-specific immunogenic proteins would be prerequisite. Since such proteins are not identified so far, our comparative approach to concomitantly analyze abscessin-, tuberculin-, and sensitin-specific T-cells is a promising alternative. In addition, marked differences in the level of T-cell response against abscessin (as compared to tuberculin and sensitin) indicated that MABSC-specific immunogenic proteins with limited cross-reactivity exist. Identification of such proteins would be an important step towards the development of a specific *in vitro* T-cell test for MABSC infection (comparable to IGRAs used for detection of *M. tuberculosis*).

Our data indicated that besides antigen-specificity, cytokine expression differed between T-cells from MABSC confirmed and non-confirmed CF patients. Cytokine expression pattern analyses revealed increased CD40L-positive / IL-2-negative T-cell proportions in confirmed MABSC infected CF patients. Evidence for CD40L as a marker of acute mycobacterial disease is provided by studies on human tuberculosis [22]. Streitz *et al*. described increased proportions of *M. tuberculosis* specific T-cells expressing CD40L in tuberculosis patients although *M. tuberculosis* specific T-cells were generally diminished in patients. Notably, CD40L expressing T-cells were optimal for discrimination of tuberculosis patients from healthy latently *M. tuberculosis* infected contacts in this study [22]. IL-2 was described previously as a marker of ‘polyfunctional’ T-cells which are crucial for protection against tuberculosis [23, 24]. We detected increased CD40L-positive / IL-2-negative MABSC-specific T-cells in CF patients with chronic MABSC infection. Therefore the presence of this MABSC specific T-cell subset may reflect the inability of these CF patients to eradicate MABSC. Possible functional deficiencies of CD40L-positive / IL-2-negative T-cells as well as causative factors for the expansion of this T-cell subset (e.g. immune evasion as described for CD8^+^ effector T-cells in tuberculosis [25]) will have to be elucidated. Furthermore, the question remains whether this T-cell subset is a general marker of increased CF patients susceptibility to recurrent pulmonary infections [26], e.g. caused by immune modulating effects of the underlying CFTR mutations [5, 27].

12 of 35 CF patients recruited in this study did not expectorate sputum samples. According to ATS criteria [11], none of these CF patients had evidence for MABSC infection. However, this assessment relies completely on the absence of clinical symptoms, which is limited by low specificity. Notably, our immune test indicated previous infection with different mycobacterial species for three sputum-negative CF patients. None of these immune responses was MABSC-dominant and cytokine expression patterns suggested no persistent infection (as compared to MABSC-confirmed CF patients). Obviously this thesis cannot be confirmed without invasive diagnostics (e.g. bronchoalveolar lavage) followed by mycobacterial culture and probably direct PCR analysis of sputum specimen, which is not indicated in asymptomatic patients without radiological evidence for a mycobacterial infection. Hence we were not able to prove our hypothesis in this regard. Future studies should perform long-term consecutive studies of larger CF patient’s cohorts to determine if immune tests allow identification of CF patients with early MABSC infection and to evaluate the individual risk to suffer from MABSC exacerbation.

In conclusion immunological and PCR-based assays may help to diagnose the NTM infection especially early during MABSC infection and exacerbation. This is of special importance for the large group of patients not able to expectorate sputum. Our novel tests may help to decide whether additional diagnostics (e.g. bronchoalveolar lavage) should be performed. As a consequence the complicated treatment of MABSC infection can be started earlier with likely better efficacy and prognosis.

## Supporting Information

S1 Fig Flow cytometry results from a MABSC-negative CF patient. A representative analysis of flow cytometry results from a MABSC-negative CF patient is depicted. Results of stimulation with different mycobacterial antigens, i.e. abscessin of *M. abscessus* (MABSC), sensitin of *M. avium* (MAC), and tuberculin of *M. tuberculosis* (MTB), the non-clonal T-cell activator SEB (Staphylococcus enterotoxin B), and without stimulation (w/o) are shown.
(EPS)

S2 Fig CD40L and IL-2 expression of M. abscessus stimulated T cells. Flow cytometry dot plots indicating for confirmed MABSC infected CF patients (CF_MABSC_) and non-confirmed MABSC T-cell responders (CF_non-confirmed_) are shown. Memory T cells are gated as indicated in Fig. 3. Proportions of memory T cells expressing CD40L, IL-2, and both are given.
(EPS)
